# Supplementary material for: Comparison of the Single-Cell Immune Landscape of Testudines from Different Habitats
Source: Cells. 2022 Dec 12;11(24):4023. doi: 10.3390/cells11244023 (PMC9816942; doi:10.3390/cells11244023)
Supplement: Supplementary file 1 [file cells-11-04023-s001.zip › Supplementary material.pdf]

**Supplementary Figure S1.**

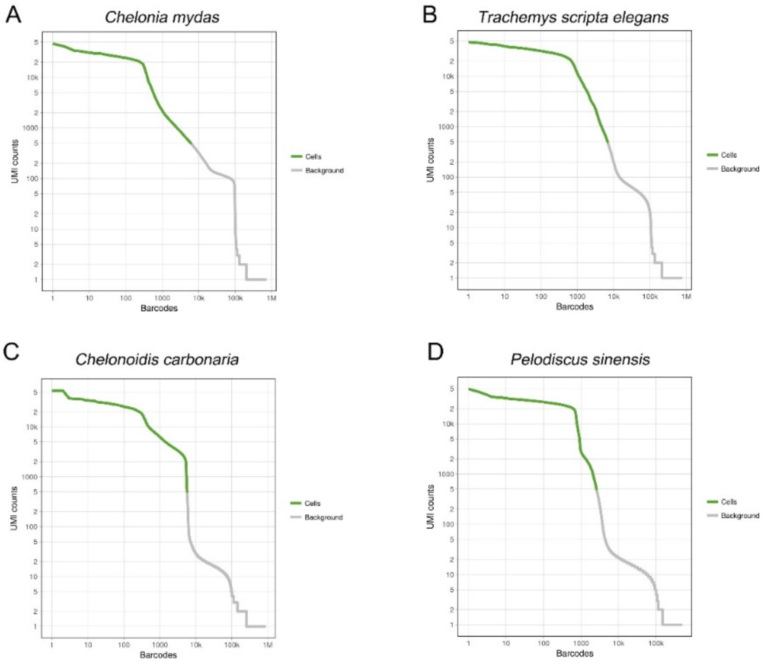


Figure S1. Map of valid cell identification

**Supplementary Figure S2.**

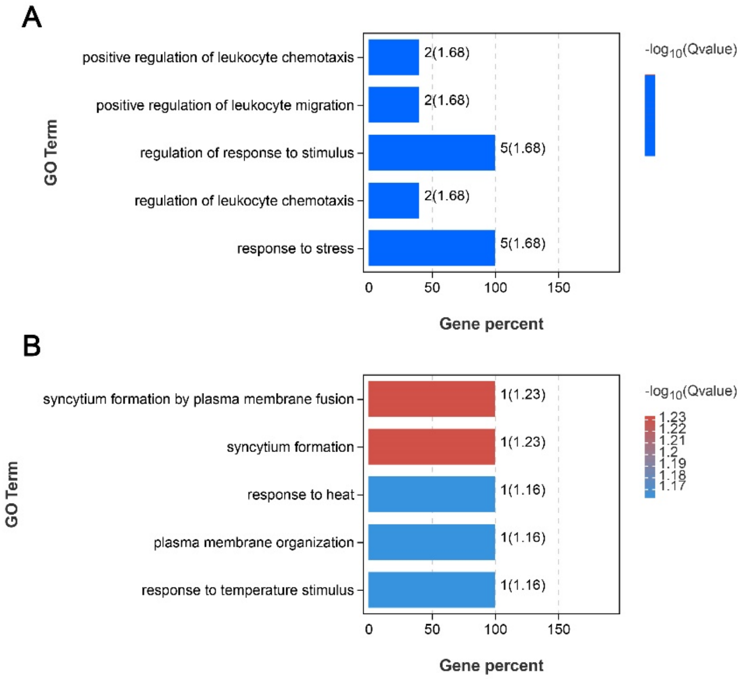


Figure S2. GO enrichment analysis. (A): LGALS1, LY86, PLBD1, MYOF, BPI, and CSF1R genes; (B): MYOF.

**Supplementary Table S1.** Morphological characteristics of testudines

| Species | Carapace length (cm) | Carapace width (cm) | Body weight (kg) |
|---|---|---|---|
| *Chelonia mydas* | 74.2±5.2 | 65.7±6.0 | 43.1±5.8 |
| *Trachemys scripta elegans* | 24.5±1.6 | 17.8±1.2 | 2.2±0.3 |
| *Chelonoidis carbonaria* | 19.9±0.7 | 12.9±0.2 | 1.5±0.1 |
| *Pelodiscus sinensis* | 22.8±0.7 | 19.2±1.0 | 1.6±0.2 |

**Supplementary Table S2.** Number of cells in each sample before and after filtration

| Species | Retained | Excluded |
|---|---|---|
| *Chelonia mydas* | 2976 | 2912 |
| *Trachemys scripta elegans* | 5739 | 965 |
| *Chelonoidis carbonaria* | 5507 | 110 |
| *Pelodiscus sinensis* | 2356 | 178 |

**Supplementary Table S3.** The results of quality control

| Species | Number of reads | Valid barcodes | Sequencing saturation | Q30 bases in barcode | Q30 bases in RNA read | Q30 bases in UMI |
|---|---|---|---|---|---|---|
| *Chelonia mydas* | 368,610,050 | 97.0% | 84.7% | 95.3% | 92.2% | 94.4% |
| *Trachemys scripta elegans* | 431,034,089 | 98.2% | 81.8% | 95.5% | 91.5% | 94.6% |
| *Chelonoidis carbonaria* | 392,723,491 | 97.8% | 81.9% | 95.6% | 92.3% | 94.8% |
| *Pelodiscus sinensis* | 443,802,858 | 98.2% | 90.0% | 95.5% | 92.0% | 94.6% |
